# High-throughput radiation sensitivity screening of 3D-head and neck squamous cell carcinoma (HNSCC) organoids using an automated radiation modulator (ARM)

**DOI:** 10.1016/j.mtbio.2026.103026

**Published:** 2026-03-19

**Authors:** Jin-Young Lee, Eunji Jeong, Sang-Yun Lee, Yu-Jeong Seong, Heejong Song, Bosung Ku, Sanghyo Kim, Wonjae Cha, Dongryul Oh, Man Ki Chung, Dong Woo Lee

**Affiliations:** a Central Research and Development Center, Medical & Bio Decision (MBD) Co., Ltd., Suwon, 16229, Republic of Korea; b Graduate School of New Drug Discovery and Development, Chungnam National University, Daejeon, 34134, Republic of Korea; c Department of Biomedical Engineering, Gachon University, Seongnam, 13120, Republic of Korea; d College of BioNano Technology, Gachon University, Seongnam, 13120, Republic of Korea; e Department of Otorhinolaryngology-Head & Neck Surgery, Seoul National University Bundang Hospital, Seoul National University College of Medicine, Seongnam-si, Gyeonggi-do, Republic of Korea; f Department of Radiation Oncology, Samsung Medical Center, Sungkyunkwan University School of Medicine, Seoul, 06351, Republic of Korea; g Department of Otorhinolaryngology-Head and Neck Surgery, Samsung Medical Center, Sungkyunkwan University School of Medicine, Seoul, 06351, Republic of Korea

**Keywords:** Radiotherapy sensitivity screening, Automated radiation modulator (ARM), High-throughput screening (HTS), Head and neck squamous cell carcinomas (HNSCC), Patient-derived organoid (PDO), Precision medicine

## Abstract

An automated radiation modulator (ARM) was developed to enable a high-throughput radiation sensitivity test using patient-derived organoids (PDOs) as an *in vitro* diagnostic device. Treatment strategies for head and neck cancer include surgery and radiotherapy. However, patient responses to radiotherapy vary widely. To overcome limitations in efficiency and scalability, the ARM was developed to provide controlled, reproducible, and high-throughput radiation delivery. Its feasibility was validated by comparing it with conventional radiation methods using two HNSCC cell lines, as well as by applying it to PDO-based radiation sensitivity tests. The ARM successfully classified head and neck cancer PDOs into radiation-sensitive and radiation-resistant groups.

OncoSensi, a radiation sensitivity screening method utilizing the ARM, was cross-validated with clinical radiotherapy outcomes, including recurrence status, in 14 patients with head and neck cancer. The multi-parameter OncoSensi model achieved a sensitivity of 80% and a specificity of 75%, demonstrating superior predictive performance compared to the single-parameter model, which yielded a sensitivity of 70% and a specificity of 50%. Statistically significant difference in recurrence-free survival (RFS) was observed between the OncoSensi-sensitive and -resistant groups. Therefore, ARM-based radiation sensitivity screening can serve as a practical tool for implementing precision medicine in radiotherapy for patients with head and neck cancer, ultimately contributing to improved treatment efficacy and patient prognosis.

## Highlights

• A novel Automated Radiation Modulator (ARM) was developed to enable high-throughput, reproducible radiation delivery for assessing radiotherapy sensitivity using patient-derived organoids (PDOs).
• OncoSensi, an ARM-based test, predicted radiotherapy outcomes in HNSCC with 80% sensitivity and 75% specificity.
• OncoSensi effectively distinguished between radiation-sensitive and -resistant PDOs, showing significant correlation with patient recurrence-free survival and supporting its clinical utility in precision radiotherapy.

## Graphical abstract

**Figure:**
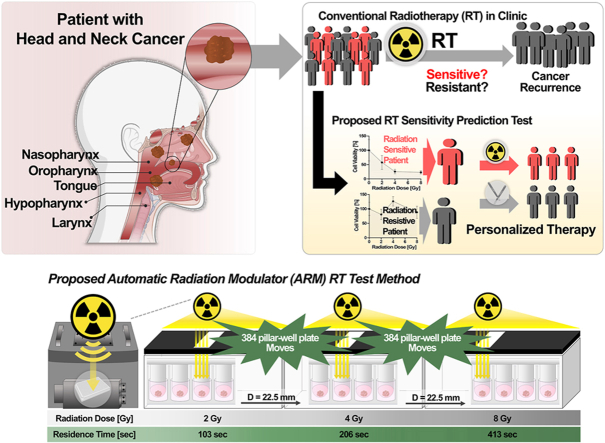

## 1 Introduction

More than 650,000 cases of head and neck cancer are diagnosed each year worldwide, with approximately 50% of patients succumbing to the disease [1,2]. In particular, more than 90% of these cancers are head and neck squamous cell carcinomas (HNSCC) [3,4]. Surgical resection has traditionally been the primary treatment modality for patients with HNSCC. Complete surgical tumor resection in patients with HNSCC often results in significant impairment of major organ function and cosmetic complications. Additionally, in advanced stages of the tumor, surgical resection alone is insufficient to achieve effective treatment [5,6]. In head and neck cancer among HNSCC, recent strategies have shifted toward an organ-preservation approach that incorporates nonsurgical treatments to enhance patients’ quality of life (QoL). Specifically, a combination of tumor resection surgery and concurrent radiotherapy (RT) is employed to eradicate the malignant tumors and improve the survival rate of patients with head and neck cancer [7,8].

Radiotherapy (RT) is an alternative treatment that can be actively used in the treatment of patients with head and neck cancer based on the decision of the clinician, contributing to improved cure rates of patients. Therefore, identifying patients who are likely respond well to RT during the early stages of treatment is crucial. Recently, precision medicine models using patient-derived organoids (PDOs) have been reported to predict RT sensitivity *in vitro* [[9], [10], [11]]. In this study, we developed an automated radiation modulator (ARM) to facilitate high-throughput radiation sensitivity screening, further expanding its clinical validation and application.

Conventionally, patient-derived organoids (PDOs) are irradiated with multiple discrete doses to quantitatively analyze the dose-dependent cytotoxic effects on cancer cells [[12], [13], [14]]. However, this approach requires repeated irradiation for each dose and cell set, resulting in low experimental throughput and substantial labor demands. Moreover, dose heterogeneity across multi-well plates represents a non-trivial and frequently encountered challenge in *in vitro* irradiation experiments, with previous study reporting dose deviations of approximately 5–10% or greater, depending on irradiation geometry, scatter conditions, and experimental setup [15]. To address these limitations, the Automated Radiation Modulator (ARM) confines irradiation to a fixed, well-characterized central field (“radiation slit”) and achieves multi-dose delivery through automated plate translation combined with dwell-time control. This design ensures that each well is exposed to an identical beam geometry under consistent scatter conditions. Using this approach, we experimentally confirmed high dose-delivery reproducibility, with a relative variation of less than 5% across 15 independent repeat runs at doses of 2, 4, 6, and 8 Gy. The ARM does not introduce a new radiation-physics principle; rather, it represents a rigorously engineered, high-throughput implementation optimized for three-dimensional organoid screening. By synchronizing automated stage motion with precisely programmed exposure intervals, the system delivers configurable multi-dose gradients across an entire 384-well plate within a single, stable irradiation cycle. A plate-level calibration workflow further ensures robust well-to-well dose reproducibility. Because the ARM can be retrofitted to standard irradiators without the need for custom beamlines, it substantially reduces operational time and cost while minimizing user intervention and plate handling. The objective of the ARM is not to establish a novel radiation delivery paradigm, but to eliminate the practical bottlenecks inherent to conventional 24- or 96-well multi-irradiation workflows, thereby enabling reproducible, high-throughput radiobiological studies at 384-well resolution. In this context, the ARM provides a practical platform for high-throughput radiation sensitivity testing using PDOs as an *in vitro* diagnostic (IVD)-compatible system. The primary aim of this study was to conduct a foundational investigation evaluating the predictive performance of organoid-based phenotypic indicators. This study does not aim to establish new genetic or metabolic marker sets; instead, it focuses on integrating reproducible, high-throughput radiation experiments with phenotype-based response modeling. The feasibility of the ARM was validated through direct comparison with conventional irradiation methods using head and neck squamous cell carcinoma (HNSCC) cell lines and head and neck cancer organoids. In addition, post-radiotherapy recurrence in head and neck cancer patients was longitudinally monitored, and the predictive performance of the ARM-based radiation sensitivity assay was assessed through comparative clinical analysis.

## 2 Materials and methods

### 2.1 Experimental procedure of radiation sensitivity screening

**Fig. 1:**
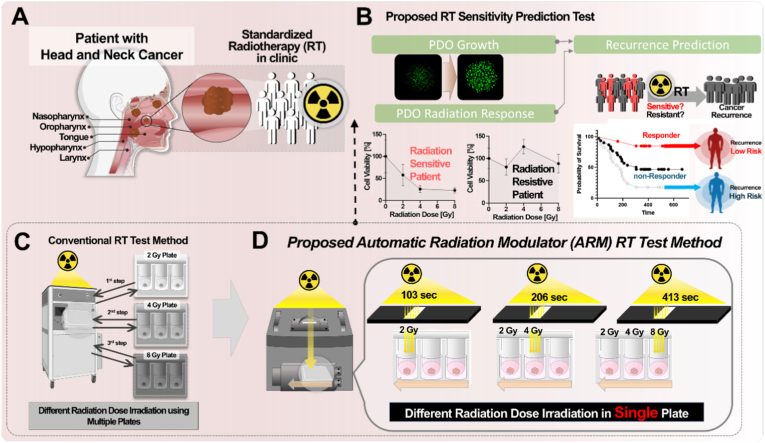
**Schematic of developed automated radiation modulator (ARM)-based radiation sensitivity screening using head and neck cancer patient-derived organoids (PDOs)** (A) Clinical patients with head and neck cancer undergo uniform radiation therapy. (B) Patients with head and neck cancer exhibit variable responses to radiotherapy, and radiation sensitivity screening can be performed in PDO models to predict efficacy in advance. (C) Previously, PDO-culture plates required repeated irradiation at individual radiation treatment doses, making the process labor-intensive. (D) To address these issues, an ARM was developed for high-throughput radiation delivery.

Patients with head and neck cancer typically receive radiotherapy (RT) as a standard treatment modality (Fig. 1A). However, clinical responses to RT vary among individuals. Therefore, if individual RT responsiveness can be predicted in advance, treatment outcomes may be maximized by selecting either radiotherapy or surgery as the primary treatment strategy based on the predicted response. Fig. 1B illustrates the multi-parameter OncoSensi screening utilizing the ARM system, which incorporates both PDO growth and radiation response to predict the risk of recurrence following RT. Traditionally, RT sensitivity screenings are performed by repeatedly exposing organoid models to varying radiation concentrations (Fig. 1C). However, this approach has limitations in implementing high-throughput screening analyses. To address this issue, we developed an ARM composed of tungsten sheets capable of shielding against radiation (Fig. 1D).

**Fig. 2:**
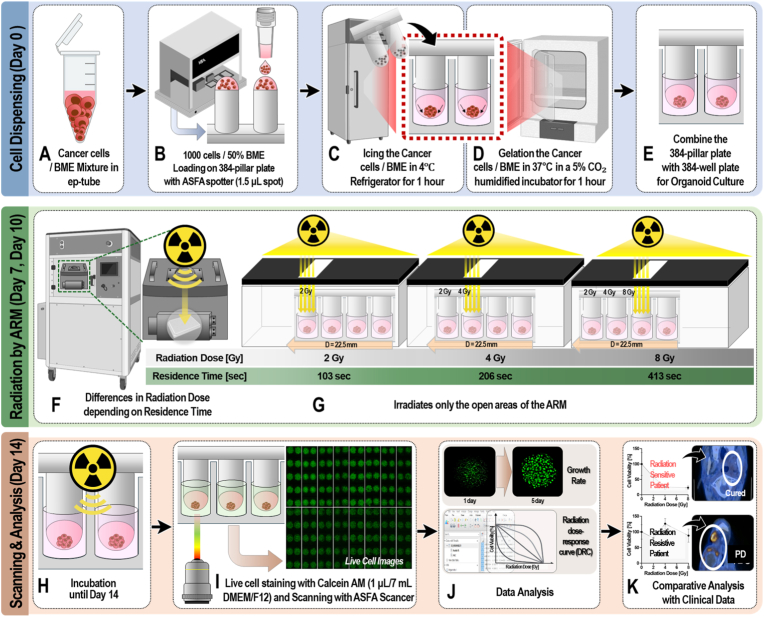
**Experimental procedures of ARM-based radiation sensitivity screening using head and neck cancer PDOs** (A) Cancer cells mixed with basement membrane extract (BME) were prepared in Eppendorf tubes. (B) The cancer cells/BME mixture was dispensed onto a 384-pillar plate using the ASFA spotter. (C) The mixture was refrigerated at 4 °C for 1 h to aggregate PDCs. (D) The loaded plate was incubated at 37 °C in a 5% CO_2_-humidified incubator for 1 h to gel the cancer cells/BME mixture. (E) The 384-pillar plate was then combined with a 384-well plate for PDO culture. (F) The ARM irradiates varying doses of radiation depending on the residence time of the plate in the radiation exposure area. Irradiation was performed on Days 7 and 10. (G) The ARM irradiates only the target areas of the organoid culture and controls precise radiation dose. (H) After irradiation, the PDOs were incubated until Day 14 to assess radiotherapy responses. (I) Live cell images were scanned to assess cell viability and analyze radiation sensitivity. (J) Radiation sensitivity was analyzed using radiation dose-response curves (DRC) based on cell viability after radiation treatment. (K) The results of the radiation sensitivity screenings were compared with clinical radiotherapy outcomes, identifying radiation-sensitive patients (cured, stable disease, SD) and radiation-resistant patients (progressive disease, PD).

HNSCC cell lines and patient-derived cells (PDCs) were used for RT sensitivity screening. The HNSCC cells separated into single cells through enzyme treatment and prepared at a concentration of approximately 1000 cells per 1.5 μL of 50% BME (50 v/v) (Fig. 2A). The cancer cells/BME mixtures were automatically dispensed onto 384-pillar plates using a 3D bioprinter (ASFA™ Spotter DN; Medical & Bio Decision, South Korea) (Fig. 2B). The 3D bioprinter dispensed 1.5 μL droplets of the cancer cells/BME mixtures on the 384-pillar plate surface. After dispensing, the pillar plate was sandwiched with an empty commercial 384-well plate and incubated at 4 °C for 1 h (Fig. 2C). This process resulted in the aggregation of cancer cells at the end of the 3D BME dome. The 384-pillar/well plate containing the cancer cell/BME mixtures were gelated at 37 °C in a 5% CO_2_-humidified incubator for 1 h (Fig. 2D). After attaching the PDC/Matrigel mixture to the surface of the 384-pillar plate through gelation, it was combined with another 384-well plate containing fresh cell culture medium and pre-cultured for one day in a 5% CO_2_-humidified incubator for 3D cultured cancer cells stabilization (Fig. 2E). The fabricated 384-pillar/well plate culturing PDOs on the top of the pillar (Fig. S1). Using the ARM, various doses of radiation were applied to the 384 pillar/well plate culturing 3D HNSCC cells (Fig. 2F). Different radiation doses ranging from 2 to 8 Gy were used, depending on the duration for which the ARM remained over the target area (Fig. 2G). The entire radiation treatment process is detailed in Video S1. After RT irradiation, the plates containing 3D cultured cells were cultured in a 5% CO_2_-humidified incubator for five days (Fig. 2H). The sensitivity of HNSCC cells to RT was quantitatively analyzed using a 3D-live cell staining method (Fig. 2I). The staining solution was prepared by adding 1 μL of Calcein AM (C1430, Invitrogen, U.S.A.) into 7 mL of DMEM/F12 medium. Cells were incubated with the staining solution for 1 h at 37 °C in a 5% CO_2_-humidified atmosphere. Afterward, the live-cell images with green fluorescence intensities (excitation/emission, 494/517 nm to lasers) were scanned using an optical scanner (ASFA Scanner, Medical and Bio Device, Korea) The viability of 3D-cells was measured by quantifying the green fluorescence area on 8-pillar strips. The area of each green fluorescence area was calculated by adding pixels with an intensity greater than 20. HNSCC cell growth rates were measured after five days of culture following RT treatment (Fig. 2J). The growth rate was calculated as the ratio of the increased area of viable cells on day five to that on day one. RT response curves were obtained by plotting cell viability (total green area) against radiation dose (0, 2, 4,8 Gy) using GraphPad Prism 10 software (Graph Pad Software, CA). The RT area under the curve (RT_AUC_) was calculated automatically in the Area Under Curve (AUC) analysis, which was performed using GraphPad Prism 10 software. The results predicted as RT-sensitive and RT-resistant through the RT sensitivity screenings were cross-validated by comparison with the RT responses of clinical patients (Fig. 2K).

### 2.2 Experimental protocol of patient-derived cell (PDC) isolation from tumor tissue with head and neck cancer

After obtaining consent from both the patient and ethics committee, tumor tissue samples were surgically removed from patients diagnosed with head and neck cancer (Fig. S2A). Clinical data were collected from the Samsung Medical Center (SMC, Seoul, Korea). The tumor tissues were transported to the laboratory and dissociated into PDCs using mechanical and enzymatic tissue dissociation protocols (Fig. S2B). After obtaining tissue measurements, tumor tissues were cut into small pieces (<2 mm) using surgical blades on a 60 mm dish containing Dulbecco's Modified Eagle's Medium/Nutrient Mixture F-12 (DMEM/F12) medium (Fig. S2C). The tumor tissue was then collected with phosphate-buffered saline (PBS) in a 50 mL conical tube and centrifuged at 1500 rpm for 3 min (Fig. S2D). After centrifugation, PBS remaining in the supernatant was removed via aspiration. The resulting tissue pellet was then resuspended in a tissue dissociation buffer (Advanced DMEM/F12 supplemented with 1% of DNase I, 10% of collagenase/hyaluronidase, and 1% penicillin–streptomycin) (Fig. S2E). Tissue dissociation buffer was added to the minced tumor tissue samples. The solution was incubated in a thermostable shaking incubator at 37 °C for 1 h. It was then centrifuged at 1500 rpm for 3 min and washed thrice with PBS to remove the enzyme solution. The cell pellet was resuspended in the organoid culture medium and filtered using a 100 μm strainer (Fig. S2F). Red blood cells (RBCs) were removed using RBC lysis buffer (Roche, Basel, Switzerland) at room temperature (RT) for 2 min (Fig. S2G). The cell/RBC lysis buffer solution was centrifuged at 1500 rpm for 3 min and washed three times with PBS to remove the enzyme solution (Fig. S2H). The extracted PDC pellet was resuspended in organoid culture medium and counted for PDO culture (Fig. S2I). The head and neck cancer organoid culture media is advanced DMEM/F12 supplemented with penicillin-streptomycin (Gibco, Grand Island, USA), glutaMAX (Gibco, Grand Island, USA), HEPES (Gibco, Grand Island, USA), R-spondin 3 (U-Protein express BV, Netherlands), Noggin (U-Protein express BV, Netherlands), Y-27632 (AdooQ, Irvine, USA), A 83-01 (AdooQ, Irvine, USA), EGF (Peprotech, Rocky Hill, USA), B-27 supplement (Gibco, Grand Island, USA), N-acetyl-L-Cystein (Sigma, St. Louis, USA), Nicotinamide (Sigma, St. Louis, USA), FGF-2 (Peprotech, Rocky Hill, USA), FGF-10 (Peprotech, Rocky Hill, USA), Prostaglandin E2 (Tocris, UK), CHIR99021 (Sigma, St. Louis, USA), Forskolin (Sigma, St. Louis, USA), and Caspofungin (Sigma, St. Louis, USA). Supplementary details are provided in Table S1.

### 2.3 Preparation and culture of HNSCC cell lines

All HNSCC cell lines (CAL-27 and FaDu) were purchased from the Korean Cell Line Bank (Seoul, South Korea) and cultured as recommended by the manufacturer. These cell lines were cultured in DMEM/F-12 medium (Gibco, Grand Island, NY) supplemented with 100 μg/mL penicillin-streptomycin (Gibco, Grand Island, USA), and 10% fetal bovine serum (Gibco, USA). Streptomycin stock solutions were used to minimize microbial contamination in the cell culture. The cell lines were maintained at 37 °C in a 5% CO_2_-humidified atmosphere and routinely passaged every four days at 70% confluence. A frozen stock was established immediately after receiving each cell line, and only early passage (<2 months) cells from the initially established frozen cell lines were used in this study. This cell line was used for 20 passages after thawing the frozen cell stock.

### 2.4 Experimental procedure of histological analysis and immunostaining

**Fig. 3:**
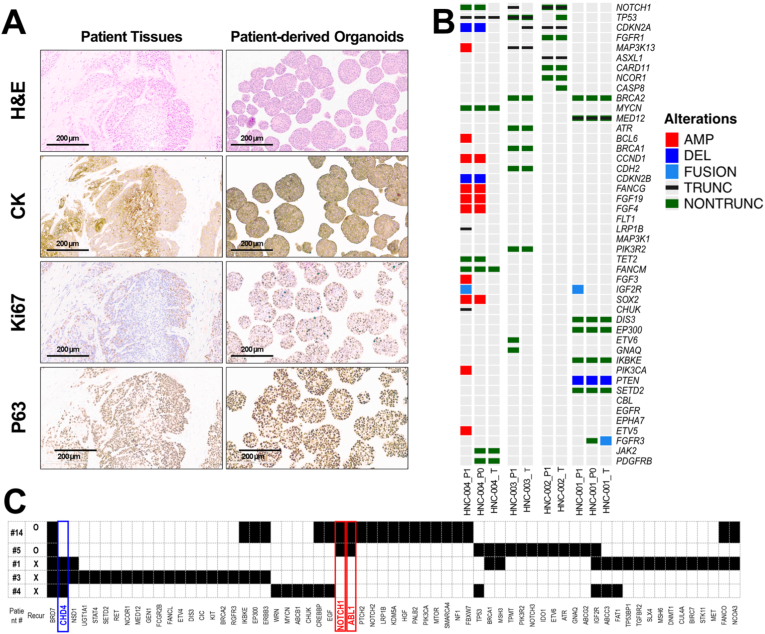
**Histological and Genetic Characterization of PDOs from head and neck cancer Patients** (A) Representative histological images of patient tissues and PDOs. Hematoxylin and eosin (H&E) staining shows the morphology of both patient tissues and PDOs. Immunohistochemistry (IHC) staining for pan-cytokeratin (CK), Ki67, and P63 reveals characteristic markers of squamous cell carcinoma in patient tissues and PDOs. Scale bars represent 200 μm. (B) Targeted sequencing analysis confirmed the genetic alterations for 377 cancer-related genes in patient tumor tissues (T) and early passaged PDOs (P0 and 1). Each column represents a specific organoid sample, and the alterations are depicted by different colored bars: amplification (AMP), deletion (DEL), fusion, truncation (TRUNC), and non-truncation (NONTRUNC). (C) Representative genetic alterations identified in three radiation-sensitive and two radiation-resistant patients.

Additional quality control (QC) samples were prepared to verify whether PDOs retained oncological characteristics similar to those of the primary tumor. The prepared PDOs for QC were subjected to histological analysis similar to that performed on the patient tissue (Fig. 3A). The isolated PDCs were seeded in a 4-well plate at a concentration of approximately 1 × 10^5^ cells and 50% Matrigel (50 v/v) per 20 μL volume. The plates were inverted and incubated at 4 °C for 5 min, then transferred and gelled for 30 min at 37 °C in a 5% CO_2_-humidified incubator. PDCs were cultured for approximately two weeks until PDOs were formed, and the culture medium was changed every three days. PDOs were fixed, embedded in paraffin, sectioned, and stained. head and neck cancer specific tumor markers, including tumor protein P53 (TP53) [16,17] and pan-cytokeratin (CK) [18,19] were stained using a Discovery XT automated immunohistochemistry stainer (Ventana Medical Systems). Hematoxylin and eosin (H&E) and immunohistochemical images were acquired using an SNC400F slide scanner (Leica, Germany). The samples were confirmed to be tumor tissues based on histopathological assessment, and the diagnosis was confirmed by a pathologist at Samsung Medical Center.

### 2.5 Comprehensive genomic analysis using high-throughput sequencing and variant detection methods

Genomic DNAs was extracted from tumor tissues of four patients with head and neck cancer and from early subcultured passage samples (passages 0 and 1) of PDOs using the QIAamp DNA Mini Kit (Qiagen, Valencia, CA, USA) (Fig. 3B). The concentration and purity of the extracted DNA were assessed using a Nanodrop 8000 UV–Vis spectrometer (Thermo Fisher Scientific) and a PicoGreen fluorescence assay with a Qubit 2.0 Fluorometer (Life Technologies). The fragment size distribution was determined using a 2200 TapeStation (Agilent Technologies, Santa Clara, CA, USA) according to the manufacturer's instructions. High-throughput sequencing was performed using the Cancer SCAN panel version 3, targeting 377 cancer-related genes as previously described [20,21]. To detect single-nucleotide variants (SNVs) with a variant allele fraction (VAF) > 1%, results from two established SNV detection methods, MuTect v1.14 and LoFreq v0.6, were integrated to enhance sensitivity. The candidate variant set was derived by combining variants identified by both callers with a high-confidence set from MuTect. Falsely detected variants from abnormally aligned, strand-biased, and clustered reads were filtered using in-house scripts including a logistic regression model trained to identify false positives in normal samples. Pindel was employed to detect indels smaller than 30 bp, whereas JuLI was used to identify deletions greater than 30 bp and structural variations (SVs) [22]. Copy number alterations in the target genes were assessed using an in-house copy number caller based on normalized copy number and B allele fractions of adjacent SNPs [20].

As shown in Fig. 3C and Table 1, quantitative growth analysis revealed that organoids #14 and #1 consistently exhibited high-growth phenotypes, whereas organoids #5, #3, and #4 showed comparatively low growth rates. Importantly, this classification was not arbitrary but was biologically consistent with their respective genomic contexts. High-growth organoids (#14 and #1) harbored genomic alterations converging on growth-related pathways, including PI3K–AKT–mTOR signaling and cell-cycle deregulation, whereas low-growth organoids (#5, #3, and #4) displayed fewer or less convergent alterations associated with proliferative signaling. Dysregulation of PI3K–AKT–mTOR signaling is a well-established driver of tumor growth, proliferation, and therapy response [23]. Although organoids #14 and #1 did not share an identical single genomic alteration, their distinct genomic profiles converged at the pathway level to support a proliferative-permissive state. For example, organoid #14 harbored alterations affecting growth factor–driven and PI3K–AKT–mTOR signaling, whereas organoid #1 was characterized by alterations in key cell-cycle and differentiation regulators such as *TP53* and *NOTCH1*. Alterations in *TP53* are known to promote uncontrolled proliferation through loss of cell-cycle checkpoint regulation [24], while aberrant *NOTCH1* signaling has been implicated in the modulation of proliferation and differentiation programs in head and neck cancers [25]. Despite these differences, both genomic contexts are recognized to promote enhanced proliferation through deregulation of cell-cycle control and activation of growth signaling pathways.

**Table 1:** Summary of the enrolled patients with head and neck cancer.

No.	Primary site	TNM Stage	HPV	Recur	RFS [days]	AUC	Z-score of AUC	Growth Rate	Z-score of Growth Rate	OncoSensi
Test Set (n = 14)										
1	Base of Tongue	cT3N0M0	Negative	No	481	535.7	0.1556	258.5	0.5761	0.712
2	Base of Tongue	cT3N2M0	Positive	No	512	337.7	−1.2579	200	0.2025	−1.738
3	Tonsil	cT3N2M0	Positive	No	539	691.1	1.2651	73.8	−1.2493	0.678
4	Oropharynx	cT2N2bM0	Negative	No	841	543.8	0.2135	129	−0.4361	−0.502
5	Oropharynx	cT2N1M0	Negative	Yes	131	657.3	1.0238	140	−0.3169	0.625
6	Tonsil	cT2N1M0	Positive	No	463	465.8	−0.3434	82.8	−1.0818	−1.702
7	Tonsil	cT2N1M0	Positive	No	795	374.9	−0.9924	262	0.5957	−0.777
8	Tonsil	cT1N1M0	Positive	No	816	565.4	0.3677	73.6	−1.2533	−0.852
9	tonsil	cT2N2M0	Positive	No	784	665	1.0788	83.5	−1.0695	0.187
10	tonsil	cT2N1M0	Positive	No	631	295.9	−1.5564	417.9	1.2756	0.036
11	tonsil	cT2N2M0	Positive	No	446	449.7	−0.4583	424.3	1.2977	1.553
12	tonsil	cT2N1M0	Positive	Yes	273	384.5	−0.9238	365.4	1.0801	0.659
13	tonsil	cT2N2M0	Negative	Yes	229	746.5	1.6606	100	−0.8069	1.122
14	Oropharynx	cT2N1M0	Negative	Yes	316	481.3	−0.2327	393	1.1861	1.539
Negative validation Set (n = 6)										
1	glottis	PT4aN1M0	-	Yes	-	719.4	1.467	152.6	−0.188	1.731
2	glottis	pT2N0M0	-	Yes	-	800	2.043	139.2	−0.321	2.335
3	glottis	pT4aN1M0	-	Yes	-	800	2.043	143.1	−0.281	2.386
4	glottis	pT4N0M0	-	Yes	-	800	2.043	131.7	−0.401	2.234
5	glottis	pT4aN0M0	-	Yes	-	634.5	0.861	260.4	0.585	1.895
6	glottis	pT4aN0M0	-	Yes	-	513.1	−0.006	500	1.530	1.926

Conversely, the genomic landscapes of low-growth organoids were comparatively less enriched for alterations linked to proliferative advantage. Collectively, these observations support the interpretation that PDO growth rate represents an integrative phenotypic output arising from heterogeneous genomic backgrounds rather than a single-marker–driven effect.

We acknowledge that detailed mechanistic analyses, such as direct assessment of PI3K/AKT activity or radiation-induced DNA damage repair capacity, would further strengthen biological interpretation; however, such investigations were beyond the scope of the present study. We therefore emphasize that our framework is not intended to replace existing molecular biomarker–based models, but rather to complement them by introducing a phenotype-aware variable that is experimentally accessible and scalable within high-throughput PDO-based radiotherapy assays. Further mechanistic and multi-omic integration studies are warranted and are recognized as important directions for future investigation.

### 2.6 Preparation of the 384-pillar plate

The 384-pillar plate was made of polystyrene-co-maleic anhydride (PS-MA) and contains 384-pillars (each with a 2 mm pillar diameter and 4.5 mm pillar-to-pillar distance). It was manufactured by plastic molding using an injection moulder (Sodic Plustech Inc., IL, USA) (Fig. 2C and Fig. S1). PS-MA, a widely used biocompatible plastic, was used to prepare 384-pillar/well plates, making it the most robust and flexible material suitable for mammalian cell culture, enzymatic reactions, viral infections, and compound screening [[26], [27], [28]]. Therefore, we successfully cultured cells mixed with various ECMs, such as alginate, Matrigel, and collagen, in 3D conditions using the aforementioned coating solution and published the experimental results [[29], [30], [31]]. To prevent contamination during cell culture and Matrigel polymerization, the surface of the pillar dishes was plasma-treated (80 W power, 5 × 10−4 Torr using air) for 10 s and coated with diluted laminin solution (L2020; 1 mg; Sigma, St. Louis, MO, USA) in PBS. To prepare the laminin-coated solution, 10 mL PBS was mixed with a 1/100 dilution of pure laminin solution (1 mg/mL). The 384-pillar plate was combined with a commercial 384-well plate to incubate PDOs for RT sensitivity prediction analysis. In addition, the 384-pillar plates were disposable consumables that were not reused to prevent cross-contamination during the experimental procedures.

### 2.7 Calculation of OncoSensi index (multiple logistic regression; MLR analysis)

In our previous studies [32,33] using lung cancer organoids, we confirmed that the growth rate of cancer organoids had a statistically significant association with the therapeutic efficacy of anticancer drugs in lung cancer patients. In the present study, we further demonstrated that the growth rate of patient-derived head and neck cancer organoids was also statistically associated with the radiotherapeutic response in head and neck cancer patients. The OncoSensi index was calculated by comprehensively considering the PDO growth rate (GR) and the AUC value, both of which were converted to Z-scores (Table 1). For all 14 patients, a multiparametric OncoSensi index was calculated using the following equation:

(1) $$OncoSensi=Z-score\left(\;\beta_{0}+\beta_{1}X_{1}+\beta_{2}X_{2}\right)$$

where *X*_*1*_ and *X*_*2*_ represent independent variables (PDOs growth rate and AUC, respectively), and coefficients *β*_*0*_*, β*_*1*_*,* and *β*_*2*_ were derived from multiple linear regression (MLR) analysis. To predict the recurrence, multiple logistic regression parameters were estimated by assigning a value of 0 for non-recurrence, and 1 for recurrence. Based on 1-year recurrence status, the OncoSensi index including AUC and Growth Rate was defined as follows:

(2) OncoSensi=Z−score[−1.313+1.324×Z−score(growth)+1.406×Z−score(AUC))]

To improve prediction accuracy, multiple logistic regression analysis was performed using AUC, growth rate, and human papillomavirus (HPV) status as parameters. HPV positivity was coded as 1 and negativity as 0. The Z-score of HPV status was calculated and incorporated into the multiple logistic regression model.

(2) OncoSensi=Z−score[−1.739+2.483×Z−score(growth)+2.411×Z−score(AUC))−0.5239×Z−score(HPV)]

### 2.8 Statistical analysis

All statistical analyses, including simple linear regression tests and *t*-tests (two-tailed), were conducted using GraphPad Prism 10 software. The goodness of fit was calculated as the R-squared (R^2^) value of a simple linear regression test. The p-value was calculated by *t*-test (two-tailed) with a confidence interval of 95%. A *p*-value of less than 0.05 was considered statistically significant.

## 3 Result and discussion

### 3.1 Preliminary genomic profiling associated with radiosensitivity patterns in head and neck cancer PDOs

The purpose of this genomic analysis is not to confirm therapeutic targets, but to explore candidate genes associated with patterns of radiosensitivity. This analysis was conducted to preliminarily investigate whether phenotypic radiosensitivity measured using ARM-based assays is associated with known molecular pathways associated with radiation response. Fig. 3C presents representative genetic alterations from three radiation-sensitive (#1, #3, #4) and two radiation-resistant (#14, #5) patients. The radiation-sensitive group harbored CHD4 mutations. The radiation-resistive group harbored NOTCH1 and ABL1. CHD4, as a core ATPase component of the NuRD chromatin remodeling complex, is rapidly recruited to DNA damage sites in response to ionizing radiation and regulates chromatin accessibility to facilitate repair factor loading [34]. Several studies in colorectal and head-and-neck cancer models have demonstrated that CHD4 overexpression correlates with radio-resistance and poor treatment response, and that CHD4 knockdown sensitizes tumor cells to ionizing radiation [35]. The NOTCH1 signaling pathway has been implicated in mediating radio-resistance in multiple cancer contexts, where increased Notch activity enhances anti-apoptotic signaling (e.g. Akt, Bcl-2) and reduces radiation‐induced cell death [36]. ABL1, a non-receptor tyrosine kinase involved in cellular stress response and DNA damage signaling, has been implicated in models of intrinsic radiosensitivity as a predictive gene [37]. These genomic findings are preliminary and hypothesis-generating. Larger cohort validation and functional studies are required to determine whether these alterations play causal roles in modulating radiation response.

### 3.2 Radiation shielding with tungsten sheet and reradiation screening using automated radiation modulator (ARM)

**Fig. 4:**
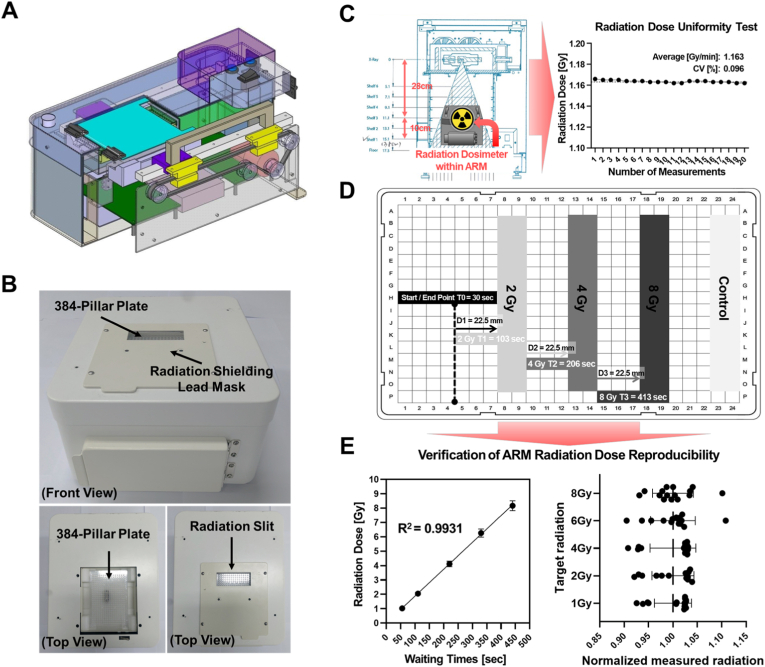
**Quantitative radiation irradiation reproducibility verification test using ARM** (A) The 3D computer-aided design (CAD) model of the radiation shielding system shows the internal components, including the radiation shielding lead mask (purple), radiation exposure area (green), and other mechanical parts. (B) Photographs of the ARM for targeted radiation exposure including the 384-pillar plate and radiation shielding lead mask. (C) Radiation dose uniformity test using a radiation dosimeter placed within the ARM. The graph shows an average radiation dose (1.163 Gy/min) and a coefficient of variation (CV) of 0.096%, indicating consistent radiation irradiation through individual measurements. (D) Measurement of radiation dose based on waiting time in the radiation exposure area. Radiation doses of 1Gy, 2 Gy, 4 Gy, and 8 Gy were quantitatively irradiated to the target plate at waiting times of 51.59, 102.98, 205.96, and 411.92 s, respectively. (E) The analysis showed that the linear correlation between radiation dose and waiting time, with an R^2^ = 0.9931, confirming that ARM radiation dose reproducibility was significant. The radiation dose variation was less than 5% across 15 independent repeat runs at 2, 4, 6, and 8 Gy. (For interpretation of the references to color in this figure legend, the reader is referred to the Web version of this article.)

We evaluated the radiation-shielding performance of the tungsten sheets constituting the ARM (Fig. S3). Radiation dose was quantitatively measured using radiation dosimeters (PTW 34001, 0.35 cc Roos® Electron Chamber, PTW Dosimetry, Germany) individually positioned in a 96-well plate (Fig. S3A). A tungsten sheet was placed in a 96-well plate containing a radiation dosimeter and irradiated using a radiation irradiator (CellRad Benchtop X-Ray Irradiator) (Fig. S3B). The dimensions of the tungsten sheet were 5 cm × 5 cm × 1.5 cm (width × length × thickness). Subsequently, radiation was applied for approximately 309 s, and the radiation dose measured using the radiation dosimeter was recorded (Fig. S3C). In the unshielded area (rows 2, 3, 10, and 11, columns A and H), a radiation dose of approximately 6 Gy was measured, whereas in the shielded area (rows 4–9, columns B–G), the radiation dose was approximately 1 Gy. The experimental results confirmed that the tungsten sheets effectively shielded against radiation. The ARM was designed using a 3D computer-aided design (CAD) method (Fig. 4A) and was subsequently fabricated as a prototype for high-throughput radiation (Fig. 4B). Next, to develop an ARM for high-throughput radiation irradiation, we first verified whether radiation consistently irradiated areas that were not shielded by the tungsten sheet in a time-dependent manner. Using a radiation dosimeter, we quantitatively measured the radiation irradiation to confirm that a constant level of radiation was applied every minute for 20 min (Fig. 4C). After 20 repetitions of the experiment over 20 min, the results showed an average radiation dose of 1.163 Gy per minute, and a coefficient of variation (CV) of 0.096% was confirmed. These results verify the effectiveness of the tungsten sheet in shielding radiation in specific areas and indicate that consistent and controlled irradiation is possible using the radiation irradiation system. Subsequently, we used the developed ARM to irradiate the radiation onto the target plate in cancer organoid cultures (Fig. 4D). The areas exposed without tungsten shielding were specifically irradiated. At this point, the organoid culture plate is automatically moved into the radiation exposure area by the ARM and irradiated accordingly. In addition, we quantitatively measured the radiation dose, which increased in a time-dependent manner (Fig. 4E). A radiation dose of 1 Gy was delivered with an exposure time of 51.59 s. As the exposure time increased, the delivered radiation dose increased proportionally. To evaluate the reproducibility of dose delivery by the ARM, radiation dose measurements were performed 15 times. A strong linear correlation between radiation exposure time and delivered dose was observed, with a high coefficient of determination (R^2^ = 0.9931). Fig. 4E also demonstrates high radiation dose-delivery reproducibility, with a relative variation of less than 5% across 15 independent repeat runs at doses of 2, 4, 6, and 8 Gy.

### 3.3 Quantitative analysis of irradiation reproducibility and radiation sensitivity screening using HNSCC cell lines

**Fig. 5:**
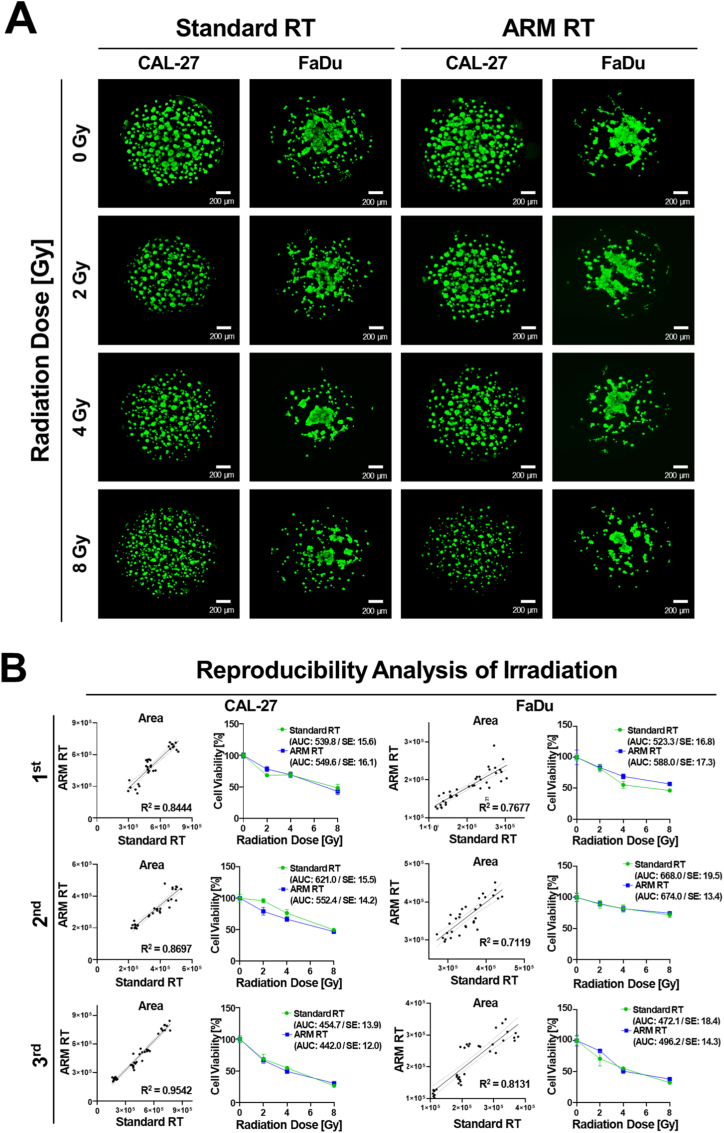
**Verification of radiation dose-dependent irradiation reproducibility using head and neck cancer cell lines** (A) 3D-live cell image analysis based on radiation dose concentrations under individual radiation treatment conditions using two head and neck cancer cell lines (CAL-27 and FaDu). (B) Graphs show cell viability measured after irradiation, with radiation sensitivity quantified by the radiation area under the curve (RT-AUC) of the radiation dose-response curves (DRCs) for CAL-27 and FaDu cell lines. The R^2^ values indicate high reproducibility for both cell lines, with values exceeding 0.7 for all conditions: CAL-27 (R^2^ = 0.8444, 0.8697, 0.9542) and FaDu (R^2^ = 0.7677, 0.7119, 0.8131). Additionally, the AUC values and standard errors (SE) for each irradiation condition showed similar levels, confirming the reproducibility of the radiation sensitivity measurements between the different irradiation methods.

Using the developed ARM, we confirmed the feasibility of quantitatively irradiating organoid culture plates using various radiation concentrations. To validate the irradiation reproducibility of the developed ARM, we cultured HNSCC lines (CAL-27 and FaDu) as organoids and quantitatively analyzed their radiation efficacy (Fig. 5). We cross-validated the conventional manual irradiation method and high-throughput irradiation method using ARM (Fig. 5A). After radiation exposure using each method, HNSCC cells were stained with a live cell-staining dye, and cell viability was analyzed based on live cell images. Biological replicate experiments were performed three times to validate the reproducibility of radiation treatment using two different HNSCC cell lines for each radiation exposure method (Fig. 5B). The experimental results using two HNSCC cell lines showed that the correlation between the cell viability values measured after radiation exposure using the conventional manual method and the developed ARM was similar, with an R-squared (R^2^) value exceeding 0.7. The quantitatively analyzed R^2^ values are listed in Table S2. Additionally, the AUC of the radiation dose-response curves (DRCs) was quantitatively measured. Analysis of the AUC and standard error (SE) values under each radiation exposure condition confirmed that the 95% confidence intervals values were comparable. The AUC and SE values for each condition are presented in Table S3. These findings confirm that the ARM can deliver radiation with the same level of precision and effectiveness as the manual method. In addition, the ARM enables high-throughput experimentation and improves the efficiency of radiation sensitivity screenings.

### 3.4 Radiation sensitivity screening of head and neck cancer PDOs using the developed ARM

**Fig. 6:**
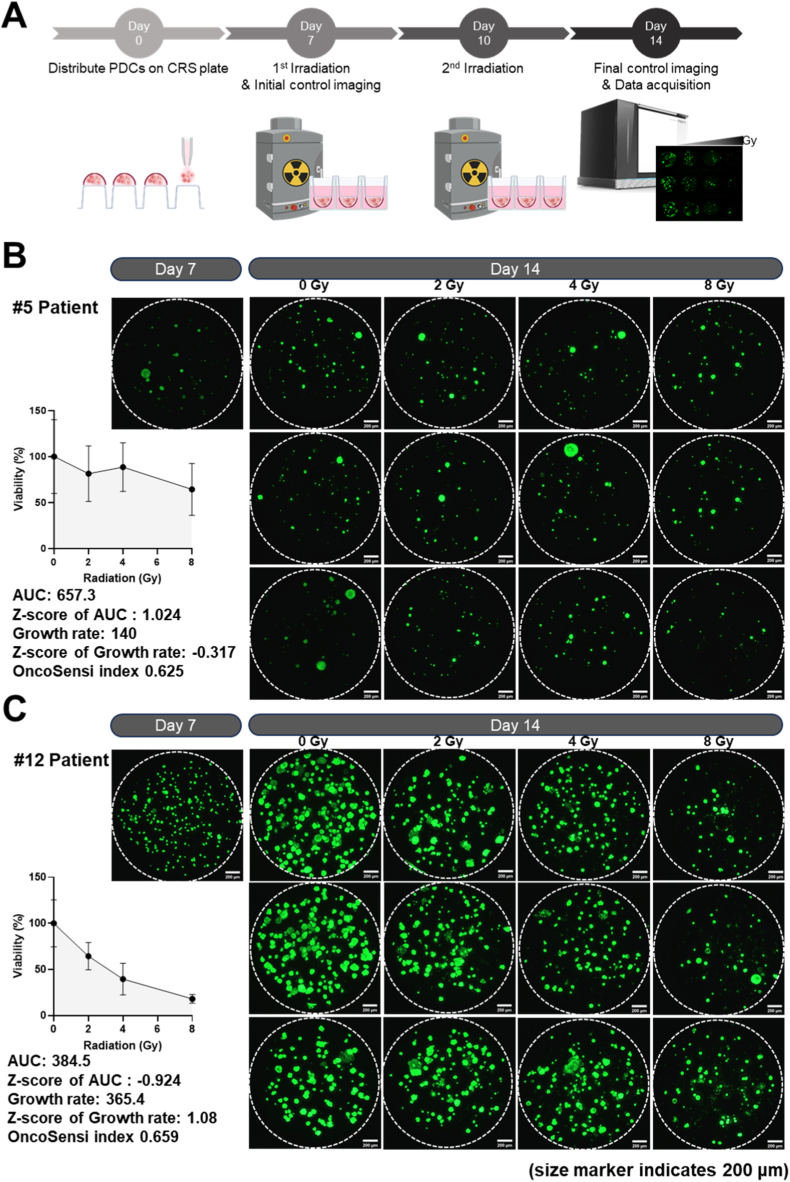
**ARM-based radiation sensitivity screening results using head and neck cancer PDOs** (A) Experimental workflow for radiation sensitivity screening using PDOs. (B) Radiation sensitivity screening of head and neck cancer PDOs from patient #5 demonstrated a radiation-resistant phenotype based on the AUC index (AUC = 1.024), indicating high resistance to radiation. Corresponding 3D live-cell images showed minimal changes in viable cell numbers across increasing radiation doses. The OncoSensi index was 0.625, also indicating a radiation-resistant phenotype. (C) In contrast, radiation sensitivity screening of head and neck cancer PDOs from patient #12 revealed a radiation-sensitive phenotype based on the AUC index. Consistent with this result, the corresponding 3D live-cell images showed marked reductions in cell viability with increasing radiation doses. Notably, although the AUC index indicated radiation sensitivity, the OncoSensi index classified this case as radiation-resistant due to the high intrinsic growth rate of the organoids.

We evaluated the performance of the developed ARM using HNSCC cell lines and confirmed that it enabled convenient and consistent radiation delivery comparable to the conventional manual method. Using the ARM, tumor cells were extracted from 76 patients with head and neck cancer, and radiation sensitivity screening was successfully performed in 43 of them. As shown in Fig. S4, clinical follow-up data were available for 14 patient samples, allowing comparison between the results of the radiation sensitivity screening and actual recurrence after radiotherapy. Among the 43 cases in which organoid-based radiation sensitivity assays were successfully performed, only 14 patients completed definitive radiotherapy and achieved a minimum clinical follow-up duration of one year. The remaining cases were excluded from outcome analysis due to referral to other institutions, refusal of radiotherapy, discontinuation of treatment, or insufficient follow-up duration. Therefore, the limited final sample size used for clinical correlation primarily reflects the stringent requirement for 1-year follow-up rather than experimental failure or selective attrition. The small number of clinically evaluable cases is a limitation of the present study. The prospective studies with larger cohorts and standardized follow-up will be required to further validate the predictive performance of the proposed framework. We isolated PDCs from head and neck cancer tumors, cultured them as PDOs, and conducted radiation sensitivity screening using the ARM (Fig. 6). In addition, we verified the similarity in tumor characteristics between early-passage head and neck cancer PDOs and the corresponding primary tumor tissues through histological and genomic analyses. The cultured PDOs exhibited well-defined cell borders and cytoplasmic keratinization, which are characteristic histological features of squamous cell carcinoma. Paraffin-embedded organoids strongly expressed pan-keratin (CK), a marker of squamous cell carcinoma, the basal cell marker P63, and the proliferation marker Ki67. Targeted sequencing of 377 cancer-related genes further confirmed that the PDOs retained the genetic characteristics of the original tumors. Among the five patients analyzed, the most common mutations were found in the NOTCH1, TP53, and CDKN2A genes. These findings suggest that the cultured PDOs accurately reflect the histological and genetic features of the patient's original tumor.

In Fig. 6A, cells were irradiated on Day 7 using the CellRad system (Precision X-ray, Inc.) with doses of 2, 4, and 8 Gy, and the same doses were administered again 3 days later. On Day 14, the viable cancer organoids were stained green. Fig. 6B and C shows two representative cases of radiation sensitivity screening conducted on head and neck cancer patient-derived organoids using the ARM. Each patient sample exhibited different cell growth rates, and the degree of decrease in cell viability and the number of viable cells varied depending on the radiation dose. Patient #5 exhibited radiation resistance (Fig. 6B), with cell viability remaining at control levels across all doses except 8 Gy, where no significant cell death was induced. Even at 8 Gy, most head and neck cancer PDOs remained viable, with only a slight reduction in organoid size observed. In contrast, patient #12, who showed a radiation-sensitive response, had a low AUC value of 384.5, indicating high sensitivity (Fig. 6C). Using the developed ARM, we were able to distinguish radiation-sensitive and radiation-resistant head and neck cancer PDOs based on live-cell staining under various radiation dose conditions. These results demonstrate that the ARM enables high-throughput screening of PDO radiation sensitivity and can contribute to predicting individual patient responses to radiotherapy.

### 3.5 Cross-validation of Radiation Sensitivity Screening with Clinical Radiotherapy Outcomes

**Fig. 7:**
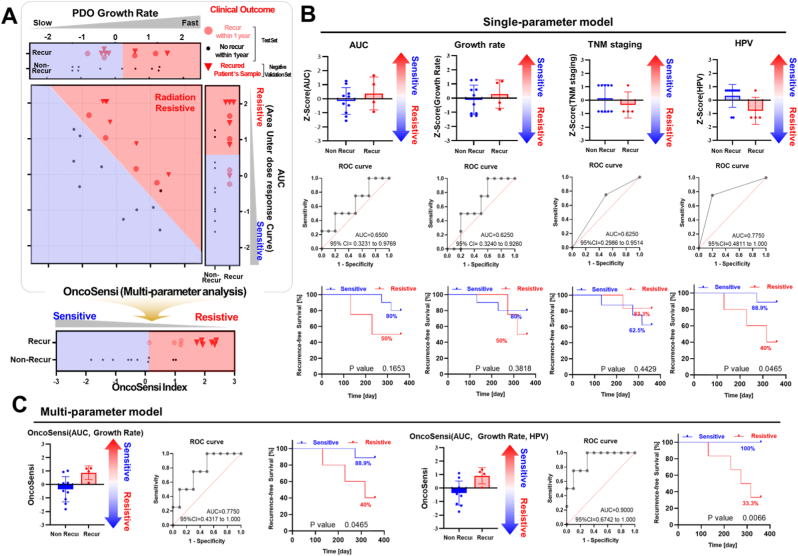
**Single and Multi-parameter Analysis for Recurrence Prediction After Radiation Therapy** Analysis of recurrence prediction indices was performed in the enrolled patients (n = 20). Among them, six patients had already experienced recurrence, and their recurrent cancer tissues were obtained to establish the variation set for OncoSensi (negative validation set). The remaining 16 patients served as the test set, from whom cancer tissues were obtained by biopsy prior to radiotherapy. Violin plots, ROC curves, and recurrence-free survival (RFS) analyses were generated using multiple indicators, including the conventional AUC, PDO growth rate, TNM stage, HPV status, and the OncoSensi index. (A) A two-parameter analysis applied to improve predictive accuracy. Patients with both high AUC and high organoid growth were classified as the strongly resistant group, whereas those with either high AUC/low growth or low AUC/high growth were categorized as the moderately resistant group. In contrast, patients with both low AUC and low organoid growth were defined as the strongly sensitive group. Among two recurrent patients who were initially predicted as sensitive group based on AUC alone, the inclusion of organoid growth rate in the OncoSensi index reclassified them as resistive group, indicating that OncoSensi (combining AUC and growth rate) provides superior predictive power for recurrence compared to AUC alone. In the negative validation set comprising six recurrent patient samples, all cases were located within the resistant region in the two-parameter analysis, whereas one patient appeared sensitive when evaluated by AUC alone. (B) Violin plots, ROC curves, and recurrence-free survival (RFS) analyses calculated using single-parameter analysis. In AUC index, AUC of ROC curve is 0.65 (95% CI = 0.3231∼0.9769). In Growth Rate index, AUC of ROC curve is 0.625 (95% CI = 0.3240∼0.9260). In TNM staging index, AUC of ROC curve is 0.625 (95% CI = 0.2986∼0.9514). In HPV index, AUC of ROC curve is 0.7750 (95% CI = 0.4811∼0.1). (C) Violin plots, ROC curves, and recurrence-free survival (RFS) analyses calculated using multi-parameter analysis. In OncoSensi(AUC, Growth Rate) index, AUC of ROC curve is 0.7750 (95% CI = 0.4317∼0.1). In OncoSensi(AUC, Growth Rate, HPV) index, AUC of ROC curve is 0.9000 (95% CI = 0.67427∼0.1).

As shown in Fig. 7, the Z-scores of individual parameters (single-parameter models) and the OncoSensi indexes (multi-parameter model) were compared between the recurrence and non-recurrence groups. Fig. 7A presents a two-dimensional plot of AUC values derived from the radiation dose–response curves and PDO growth rates, based on the 16-patient test set and the 6-patient negative validation set. Among the recurrent cases in test set, two patients exhibited low AUC values but showed high PDO growth rates. These results indicate that high AUC values combined with high PDO growth rates are associated with a higher risk of recurrence within 1 year. Therefore, to improve both sensitivity and specificity, we propose the multi-parameter OncoSensi model, which integrates AUC and PDO growth rate. In the negative validation set comprising six recurrent patient samples (Fig. 7A), all cases were located within the resistant region in the two-parameter analysis, whereas one patient appeared sensitive when evaluated by AUC alone. As shown in Fig. 7B, when using AUC alone, the AUC value of the ROC curve was 0.65, with the average Z-score being slightly higher in the recurrence group than in the non-recurrence group. When using the PDO growth rate alone, the AUC of the ROC curve was 0.625. Most patients were classified as stage III or IV, and the relationship between stage and recurrence appeared to be reversed. The recurrence-free survival (RFS) analysis showed an unexpected trend—patients categorized as the “sensitive” group exhibited lower survival than those in the resistant group. This inverse pattern likely reflects the limited number of advanced-stage cases included in the cohort. Therefore, TNM stage was excluded from the multi-parameter analysis to avoid confounding effects. HPV-positive head and neck cancer, particularly oropharyngeal carcinoma, has been consistently associated with enhanced radiosensitivity and improved survival compared to HPV-negative tumors [[38], [39], [40], [41]]. The incorporation of HPV status into the multi-parameter model is supported by previous large-scale clinical studies demonstrating improved radiotherapy response and survival in HPV-positive head and neck cancer. HPV status showed an AUC of 0.775, which was the highest among the single-parameter models. The recurrence-free survival (RFS) based on HPV status significantly distinguished the radio-sensitive and radio-resistant groups. In contrast, the OncoSensi model demonstrated significantly improved predictive performance. The AUC values of the ROC curves were 0.775 and 0.9, depending on whether HPV status was considered. The AUC of the ROC curve for the OncoSensi model incorporating AUC, growth rate, and HPV status was 0.9 (Fig. 7C), indicating high accuracy in predicting recurrence after radiotherapy. Although AUC has conventionally been used as an indicator of radiation sensitivity, our study did not show a statistically significant difference in RFS between the sensitive and resistant groups based on AUC alone (1-year RFS: 80% vs. 50%, *p* = 0.6550). In contrast, prognostic discrimination was significantly improved when survival analysis was performed using the OncoSensi index, a multi-parameter model integrating PDO growth rate. Patients classified as the sensitive group by the OncoSensi index (AUC and growth rate) showed significantly better outcomes than those classified as the resistant group (1-year PFS: 88.9% vs. 40.0%, *p* = 0.0469), supporting the clinical utility of the OncoSensi index. Moreover, the OncoSensi model incorporating AUC, growth rate, and HPV status showed the best prognostic performance (1-year PFS: 100% vs. 33.3%, *p* = 0.0066).

**Fig. 8:**
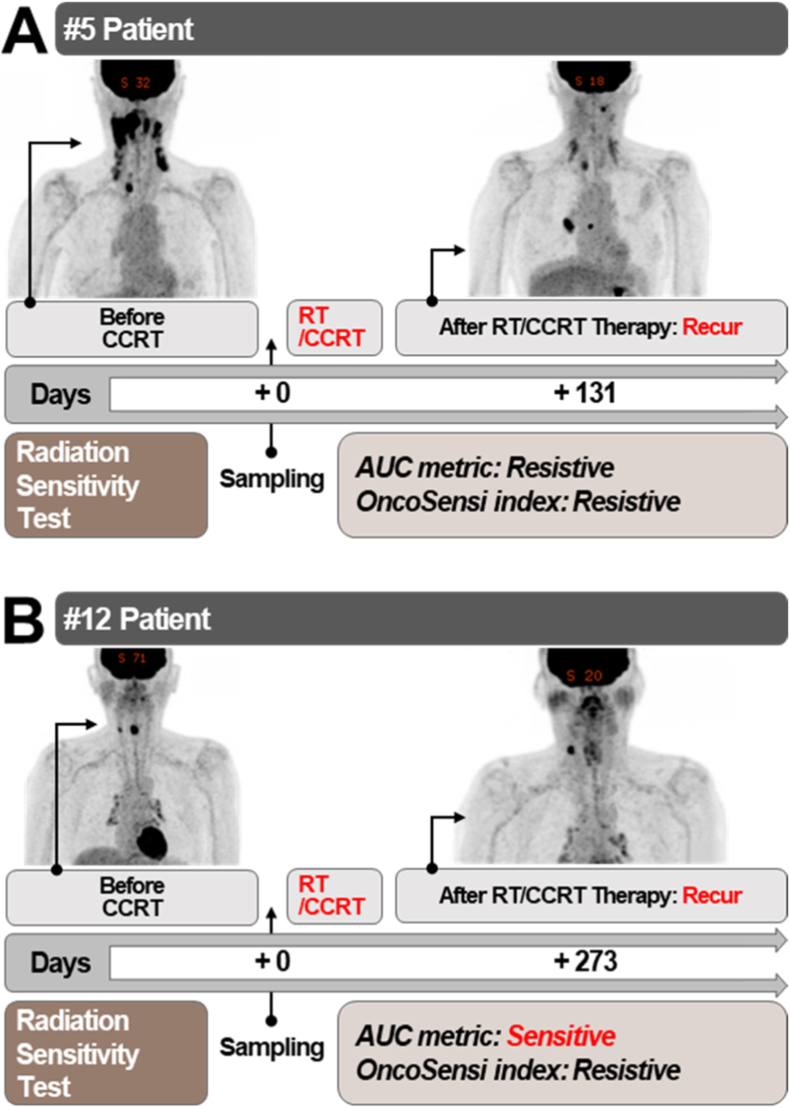
Cross-validation of radiation sensitivity screening results with clinical radiotherapy outcomes in patients with head and neck cancer **(A)** Radiation sensitivity screening indicated a radiation-resistant phenotype in patient #5. Clinical follow-up after surgery and radiotherapy confirmed cancer recurrence and progression, consistent with both the AUC and OncoSensi indices. **(B)** Radiation sensitivity screening indicated a radiation-sensitive phenotype based on the AUC index but a radiation-resistant phenotype based on the OncoSensi index in patient #12. Clinical follow-up after surgery and radiotherapy confirmed disease progression (PD), which was consistent with the OncoSensi index but not with the AUC index.

Patient responses to radiotherapy were clinically evaluated by oncologists according to the RECIST 1.1 (Response Evaluation Criteria in Solid Tumors) guidelines [42,43]. RECIST evaluates tumor burden based on the sum of the maximum diameters of target lesions observed on imaging. The criteria are defined as follows: complete or partial response (CR/PR), indicating complete disappearance or a decrease in lesion size of more than 30%; stable disease (SD), defined as a change between −30% and +20%; and progressive disease (PD), defined as an increase in lesion size of more than 20%. In this study, PD was interpreted as recurrence after radiotherapy. A comparative analysis of clinical treatment outcomes was conducted. In #5 patient was classified as resistive based on Z-scores above the cutoff using both AUC index and OncoSensi index. (Fig. 8A). However, in #12 patient, recurrence occurred, but there was a discrepancy between predictions from AUC index and the OncoSensi index. While AUC classified #12 patient as a sensitive, the OncoSensi index identified the patient as resistive. Clinically, #12 patient experienced recurrence after first-line chemotherapy (Fig. 8B). Those patients experienced recurrence after radiation therapy. These prediction results were consistent with actual clinical outcomes, further supporting the superior clinical utility of the OncoSensi index compared to the AUC index. By identifying patients who are likely to respond to radiotherapy through radiation sensitivity screening, unnecessary radiation exposure in radiation-resistant patients can be avoided. This approach helps minimize treatment-related side effects and improves patients’ quality of life (QoL) through more effective and individualized RT. For patients predicted to be resistant to RT, alternative treatment strategies such as surgery or targeted drug therapies should be considered as priorities. This strategy not only reduces the risk of radiation-induced side effects but also enhances the therapeutic efficacy of other available treatment options. However, this strategy requires prospective validation of treatment selection based on the screening results (e.g., whether patients who receive radiotherapy or surgery according to OncoSensi predictions exhibit better prognoses). Such validation was not performed at this research stage, as it would require direct clinical intervention in patient treatment, posing significant ethical and financial constraints. Specifically, this would involve substantial clinical costs related to patient risk management, insurance coverage, and treatment expenses, which are beyond the scope of the present study.

## 4 Conclusions

In this study, we developed an Automated Radiation Module (ARM) for high-throughput screening of radiation sensitivity in patient-derived organoids (PDOs). The ARM demonstrated performance comparable to conventional manual irradiation methods, while providing enhanced efficiency, reproducibility, and control. Furthermore, we confirmed the potential of PDO-based radiation sensitivity screening as a predictive tool for clinical outcomes.

The multi-parameter OncoSensi model utilizing the ARM achieved a sensitivity of 80% and a specificity of 75% based on recurrence follow-up data from 14 patients with head and neck cancer, demonstrating superior predictive performance compared to the single-parameter model, which showed a sensitivity of 70% and specificity of 50%. In addition, a statistically significant difference in recurrence-free survival (RFS) was observed between the OncoSensi-sensitive and -resistant groups. Therefore, ARM-based radiation sensitivity screening can serve as a practical tool for implementing precision medicine in radiotherapy for patients with head and neck cancer, ultimately contributing to improved treatment efficacy and patient prognosis. This model can be applied as an *in vitro* diagnostic device for assessing radiation sensitivity in oropharyngeal cancer and may further improve the accuracy of radiotherapy response prediction when combined with genomic markers for head and neck cancer in the future. The applicability of OncoSensi in specific clinical contexts such as re-irradiation or concurrent chemoradiotherapy was also not addressed in the present study. These scenarios require distinct treatment paradigms and longer-term validation and will be important directions for future clinical investigations.

## Ethics approval and consent to participate

This study was conducted in accordance with the ethical standards of the Declaration of Helsinki and national and international guidelines. It was approved by the Institutional Review Board of the SAMSUNG Medical Center (IRB No. 2020-09-002-007). Written Consent was obtained from all participants, and minors were excluded from the study.

## CRediT authorship contribution statement

**Jin-Young Lee:** Data curation, Formal analysis, Investigation, Methodology, Resources, Validation, Visualization. **Eunji Jeong:** Conceptualization, Data curation, Formal analysis, Methodology, Resources, Validation, Visualization, Writing – original draft. **Sang-Yun Lee:** Conceptualization, Data curation, Formal analysis, Funding acquisition, Investigation, Methodology, Resources, Validation, Visualization, Writing – original draft, Writing – review & editing. **Yu-Jeong Seong:** Data curation, Visualization. **Heejong Song:** Conceptualization, Data curation, Visualization. **Bosung Ku:** Resources, Supervision. **Sanghyo Kim:** Conceptualization, Data curation. **Wonjae Cha:** Resources, Visualization. **Dongryul Oh:** Resources, Supervision. **Man Ki Chung:** Conceptualization, Data curation, Formal analysis, Investigation, Methodology, Project administration, Resources, Supervision, Validation, Writing – original draft, Writing – review & editing. **Dong Woo Lee:** Conceptualization, Data curation, Formal analysis, Funding acquisition, Investigation, Methodology, Project administration, Resources, Supervision, Validation, Visualization, Writing – original draft, Writing – review & editing.

## Declaration of competing interest

The authors declare that they have no known competing financial interests or personal relationships that could have appeared to influence the work reported in this paper.

## Data Availability

Data will be made available on request.
