# Supplementary material for: High-throughput radiation sensitivity screening of 3D-head and neck squamous cell carcinoma (HNSCC) organoids using an automated radiation modulator (ARM)
Source: Mater Today Bio. 2026 Mar 19;38:103026. doi: 10.1016/j.mtbio.2026.103026 (PMC13053779; doi:10.1016/j.mtbio.2026.103026)
Supplement: Multimedia component 1 [file mmc1.docx]

Supplementary Information

**High-Throughput Radiation Sensitivity Screening of
3D-Head and Neck Squamous Cell Carcinoma (HNSCC) Organoids
Using an Automated Radiation Modulator (ARM)**

Jin-Young Lee^a,‡^, Eunji Jeong^a,‡^, Sang-Yun Lee^b,‡^, Yu-Jeong Seong^c^, Heejong Song^a^, Bosung Ku^a^, Sanghyo Kim^d^, Wonjae Cha^e^, Dongryul Oh^f^, Man Ki Chung^g*^, Dong Woo Lee^c*^

^a^ Central Research and Development Center, Medical & Bio Decision (MBD) Co., Ltd., Suwon, 16229, Republic of Korea

^b^ Graduate School of New Drug Discovery and Development, Chungnam National University, Daejeon, 34134, Republic of Korea

^c^ Department of Biomedical Engineering, Gachon University, Seongnam, 13120, Republic of Korea

^d^ College of BioNano Technology, Gachon University, Seongnam, 13120, Republic of Korea

^e^ Department of Otorhinolaryngology-Head & Neck Surgery, Seoul National University Bundang Hospital, Seoul National University College of Medicine, Seongnam-si, Gyeonggi-do, Republic of Korea

^f^ Department of Radiation Oncology, Samsung Medical Center, Sungkyunkwan University School of Medicine, Seoul, 06351, Republic of Korea

^g^ Department of Otorhinolaryngology-Head and Neck Surgery, Samsung Medical Center, Sungkyunkwan University School of Medicine, Seoul 06351, Republic of Korea

‡These authors contributed equally to this work: Jin-Young Lee, Eunji Jeong and Sang-Yun Lee.

* Corresponding Author

Man Ki Chung, MD, Ph.D., Department of Otorhinolaryngology-Head and Neck Surgery, Samsung Medical Center, Sungkyunkwan University School of Medicine, Seoul 06351, Republic of Korea. E-mail: manki.chung@gmail.com

Dong Woo Lee, Ph.D., Department of Biomedical Engineering, Gachon University, Seongnam, 13120, Republic of Korea. E-mail: dw2010.lee@gmail.com

**Supplementary Files**

**
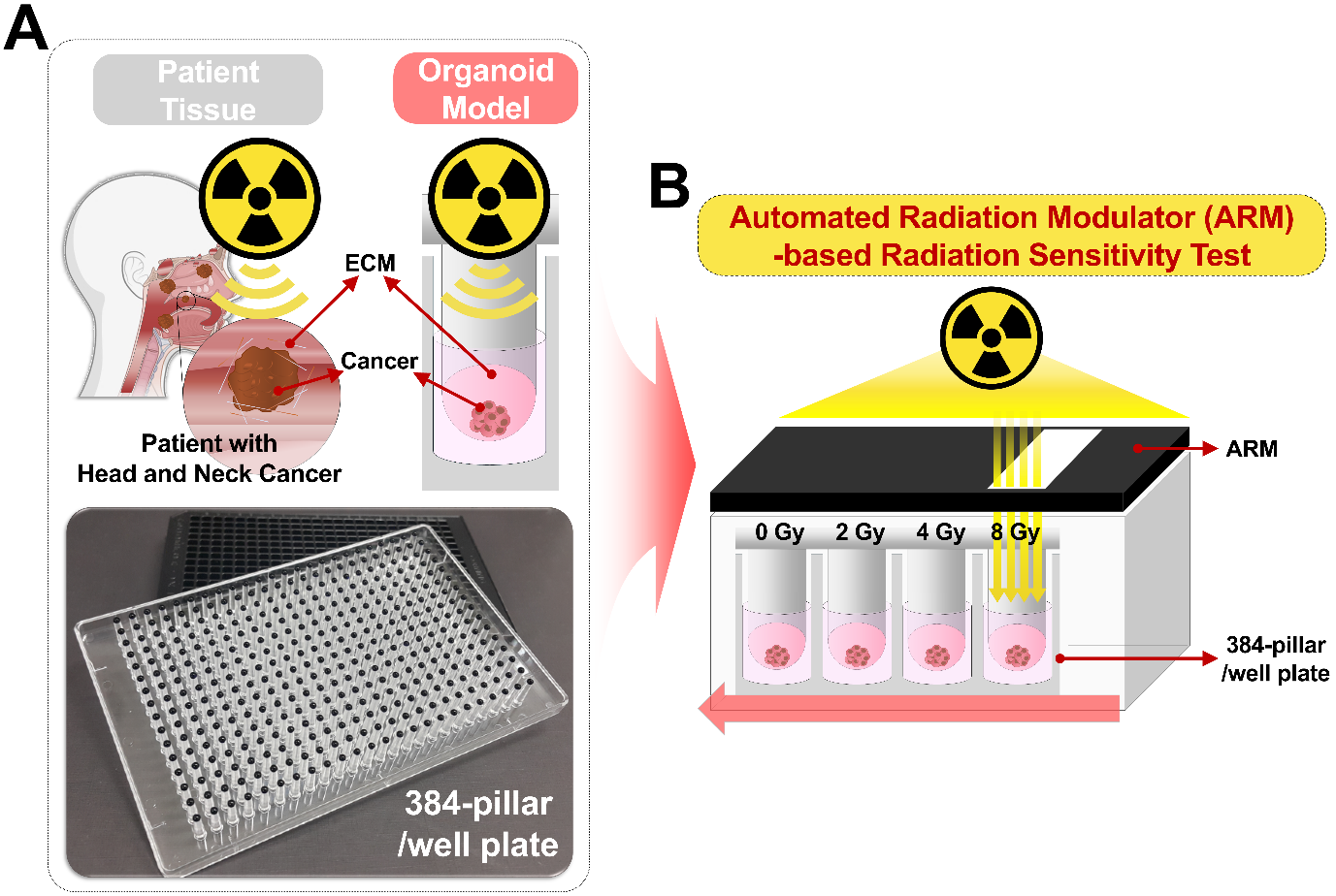
**

**Fig. S1 384-pillar/well plate for ARM-based radiation sensitivity screening**

(A) Irradiation was performed using head and neck cancer organoid models cultured in 384-Pillar plates in the same manner as patient tumor tissue. (B) Performing automated high-throughput radiation irradiation and radiation sensitivity screening analysis using ARM


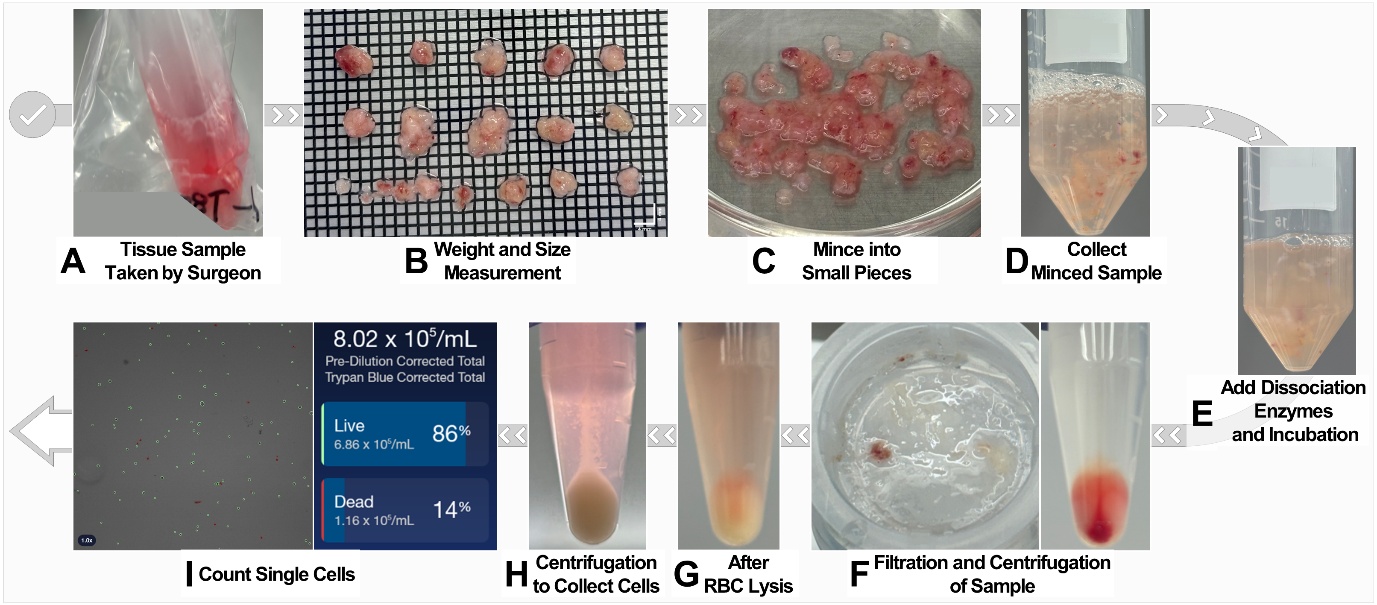


**Fig. S2. Experimental process for isolating patient-derived cells from tumor tissue of patients with head and neck cancer**

(A) Tissue sample obtained from a head and neck cancer patient by the surgeon. (B) The tissue is weighed and measured for size. (C) The sample is minced into small pieces. (D) Minced tissue is collected into a tube. (E) Dissociation enzymes are added to the minced tissue, followed by incubation. (F) Filtration and centrifugation are performed to process the sample. (G) After red blood cell (RBC) lysis, the sample is prepared for further analysis. (H) Post-RBC lysis, the sample is prepared for further processing. (H) Counting of live and dead cells using an automatic cell counter, with the total viable cell concentration recorded.

**
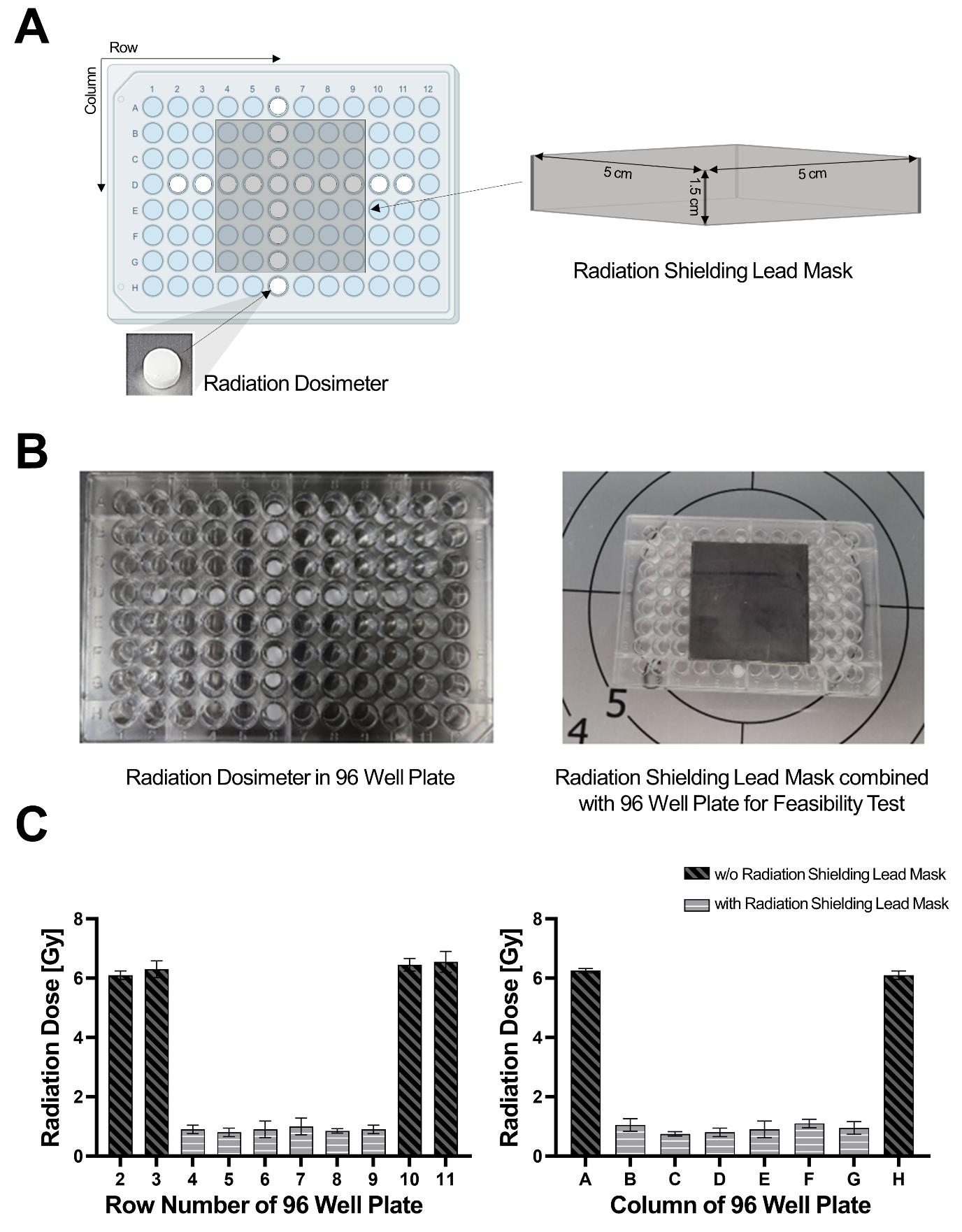
**

**Fig. S3 Measurement of penetrated radiation dose according to difference in tungsten sheet thickness**

(A) Schematic representation of the experimental setup using a 96-well plate for radiation dosimetry. The layout shows the placement of radiation dosimeters in the wells of the plate, and the radiation shielding lead mask dimensions (5 cm × 5 cm × 15 cm) are illustrated. (B) Photographs of the experimental setup: on the left, radiation dosimeters placed in a 96-well plate; on the right, a radiation shielding lead mask combined with the 96-well plate for feasibility testing. (C) Radiation dose measurements (Gy) for different rows and columns of the 96-well plate. Data are shown for plates with and without the radiation-shielding lead mask. The graphs illustrate the variation in radiation dose across different rows and columns, indicating the shielding effectiveness.


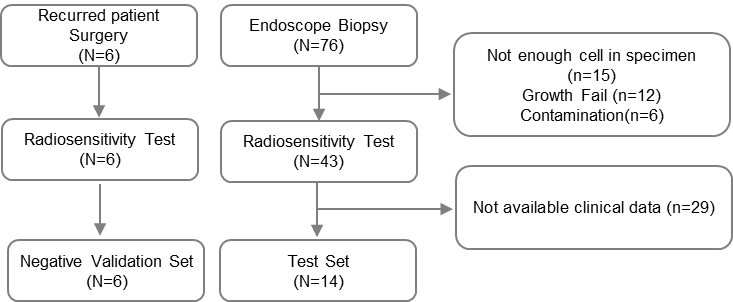


**Fig. S4 Flowchart of patient enrollment and classification**

A total of 76 head and neck cancer patient tissue samples were collected for clinical trial use. Among these, 15 samples did not yield viable cancer cells, resulting in 43 samples eligible for drug screening analysis. Of these, 29 samples lacked sufficient clinical follow-up data. As a result, 14 samples (Test Set) were included in the final analysis. six patients had already experienced recurrence, and their recurrent cancer tissues were obtained to establish the validation set for OncoSensi (negative validation set).

**Table S1 Detailed composition of the head and neck cancer patient-derived organoids culture medium.**

| **Components** | **Source** | **Catalog. No** | **Final Concentration** |
| --- | --- | --- | --- |
| **Advanced DMEM/F-12** | Gibco | 12634028 | 1 X |
| **Penicillin-streptomycin** | Gibco | 15140-122 | 100 µg/ml |
| **GlutaMAX 100X** | Gibco | 35050-061 | 1 X |
| **HEPES(1M)** | Gibco | 15630-056 | 10 mM |
| **R-spondin 3 CM** | U-Protein express BV | R001 | 4 % |
| **Noggin CM** | U-Protein express BV | N002 | 4 % |
| **Y-27632 (Rock inhibitor)** | AdooQ | A11001 | 10 µM |
| **A83-01** | AdooQ | A12358 | 500 nM |
| **EGF** | Peprotech | AF-100-15 | 50 ng/ml |
| **B-27 supplement (50X)** | Gibco | 17504044 | 1 X |
| **N-Acetyl-L-cysteine** | Sigma-Aldrich | A9165-5G | 1.25 mM |
| **Nicotinamide** | Sigma-Aldrich | N0636 | 10 mM |
| **Recombinant Human FGF-2** | Peprotech | 100-18B | 5 ng/ml |
| **Recombinant Human FGF-10** | Peprotech | 100-26 | 10 ng/ml |
| **Prostaglandin E_2_** | Tocris | 2296 | 1 μΜ |
| **CHIR99021** | Sigma-Aldrich | SML 1046 | 0.3 μΜ |
| **Forskolin** | Sigma-Aldrich | F6886 | 1 μΜ |
| **Caspofungin** | Sigma-Aldrich | SML 0425 | 0.5 µg/ml |

**Table S2 Correlation analysis results under individual radiation irradiation method conditions**

| **R-squared values**  **(Standard RT vs ARM RT)** | **CAL-27** | **FaDu** |
| --- | --- | --- |
| 1^st^ Experiment | 0.8444 | 0.7677 |
| 2^nd^ Experiment | 0.8697 | 0.7119 |
| 3^rd^ Experiment | 0.9542 | 0.8131 |

**Table S3 Quantitative analysis results of radiation efficacy according to individual radiation irradiation method conditions**

| **Experiment** | **CAL-27** | | | | **FaDu** | | | |
| --- | --- | --- | --- | --- | --- | --- | --- | --- |
|  | **Standard RT** | | **ARM RT** | | **Standard RT** | | **ARM RT** | |
|  | **AUC** | **SE** | **AUC** | **SE** | **AUC** | **SE** | **AUC** | **SE** |
| 1^st^ Experiment | 539.8 | 15.6 | 549.6 | 16.1 | 523.3 | 16.8 | 588.0 | 17.3 |
| 2^nd^ Experiment | 621.0 | 15.5 | 552.4 | 14.2 | 668.0 | 19.5 | 674.0 | 13.4 |
| 3^rd^ Experiment | 454.7 | 13.9 | 442.0 | 12.0 | 472.1 | 18.4 | 496.2 | 14.3 |

**Supplementary Video 1. Summary video clip of the radiation sensitivity screening procedure using the developed ARM**
